# Factors and a model to predict three-month mortality in patients with acute fatty liver of pregnancy from two medical centers

**DOI:** 10.1186/s12884-023-06233-w

**Published:** 2024-01-04

**Authors:** QiaoZhen Peng, TeXuan Zhu, JingRui Huang, YueLan Liu, Jian Huang, WeiShe Zhang

**Affiliations:** 1 Department of Obstetrics, Xiangya Hospital, Central South University, Hunan, Changsha, 410008 China; 2 Department of Obstetrics and Gynecology, the Third Affiliated Hospital of Zhengzhou University, Henan, Zhengzhou, 450052 China; 3 Department of Obstetrics and Gynecology, the Second Xiangya Hospital, Central South University, Hunan, Changsha, 410011 China

**Keywords:** Acute fatty liver of pregnancy, Prognostic factor, Mortality, Outcome

## Abstract

**Background:**

Acute fatty liver of pregnancy (AFLP) is an uncommon but potentially life-threatening complication. Lacking of prognostic factors and models renders prediction of outcomes difficult. This study aims to explore factors and develop a prognostic model to predict three-month mortality of AFLP.

**Methods:**

This retrospective study included 78 consecutive patients fulfilling both clinical and laboratory criteria and Swansea criteria for diagnosis of AFLP. Univariate and multivariate cox regression analyses were used to identify predictive factors of mortality. Predictive efficacy of prognostic index for AFLP (PI-AFLP) was compared with the other four liver disease models using receiver operating characteristic (ROC) curve.

**Results:**

AFLP-related three-month mortality of two medical centers was 14.10% (11/78). International normalised ratio (INR, hazard ratio [HR] = 3.446; 95% confidence interval [CI], 1.324–8.970), total bilirubin (TBIL, HR = 1.005; 95% CI, 1.000-1.010), creatine (Scr, HR = 1.007; 95% CI, 1.001–1.013), low platelet (PLT, HR = 0.964; 95% CI, 0.931–0.997) at 72 h postpartum were confirmed as significant predictors of mortality. Artificial liver support (ALS, HR = 0.123; 95% CI, 0.012–1.254) was confirmed as an effective measure to improve severe patients’ prognosis. Predictive accuracy of PI-AFLP was 0.874. Area under the receiver operating characteristic curves (AUCs) of liver disease models for end-stage liver disease (MELD), MELD-Na, integrated MELD (iMELD) and pregnancy-specific liver disease (PSLD) were 0.781, 0.774, 0.744 and 0.643, respectively.

**Conclusion:**

TBIL, INR, Scr and PLT at 72 h postpartum are significant predictors of three-month mortality in AFLP patients. ALS is an effective measure to improve severe patients’ prognosis. PI-AFLP calculated by TBIL, INR, Scr, PLT and ALS was a sensitive and specific model to predict mortality of AFLP.

## Background

Acute fatty liver of pregnancy (AFLP) is an uncommon but potentially life-threatening perinatal complication [1]. Even with modern treatment, mortality of AFLP can still reach 12-15% [2, 3]. Moreover, risk factors for AFLP-related mortality are not well established. Up to now, lacking of prognostic factors or models for AFLP renders prediction of clinical outcomes difficult. We aimed to explore prognostic factors and develop a more accurate model to predict mortality of AFLP.

So far, there are four scoring systems designed for hepatic failure as potential models to predict outcomes of AFLP. Model for end-stage liver disease (MELD) consisting of serum bilirubin and creatinine levels, international normalized ratio (INR) for prothrombin time, and etiology of liver disease was validated in patients stratified according to different causes of liver disease, such as acute liver failure and liver cirrhosis of various causes [4, 5]. In recent years, some scholars have also used it to evaluate prognosis of liver disease during pregnancy [6, 7]. Subsequently, serum sodium levels were incorporated into MELD to form the MELD-Na model, which provides more accurate survival predictions than MELD alone [8, 9]. Similarly, MELD incorporating with serum sodium levels and patients’ age (integrated MELD, iMELD) also performed better than original MELD in predicting 3-, 6- and 12-month mortality in patients with cirrhosis [10].

Murali et al. developed a model to predict 1-month mortality in patients with pregnancy-specific liver disease (PSLD) [11]. PSLDs mainly include preeclampsia, AFLP and haemolysis, elevated liver enzymes and low platelet count (HELLP) syndrome. And the area under the receiver operating characteristic curve (AUC) of this new model to predict 1-month mortality of PSLDs is 0.86. However, we found that this new PSLD model did not accurately predict outcomes of AFLP patients in our hospital. Moreover, the three PSLDs listed above differed not only morbidity and mortality but also pathogenesis, clinical manifestations and risk factors for death [2, 12]. Therefore, a generalised model cannot include relevant high-risk factors for all PSLDs.

In addition to the above scoring systems for end-stage liver disease, several general scoring systems are used to determine prognosis of severely ill patients, such as Sequential Organ Failure Assessment (SOFA) and Acute Physiology and Chronic Health Evaluation II (APACHE II) scoring systems [13, 14]. However, the prognostic efficiency of these scoring systems is diminished when they are applied to specific subpopulation of severely ill patients. Thus, a model based on a general scoring system may not be as accurate as one based on a specific scoring system.

Hence, we aim to search for relevant information to explore factors related to AFLP outcomes and to develop a new prognostic model to accurately predict mortality of AFLP. The new prognostic model will be based on the clinicopathological features of AFLP. We also intend to compare the predictive values of all five models (the new model, MELD, MELD-Na, iMELD and PSLD model) to determine which one best predicted clinical outcome.

## Methods

We performed a retrospective study of 99 consecutive patients who were suspected to have AFLP admitted to Xiangya Hospital and the Second Xiangya Hospital, Central South University, Hunan, China between November 2009 and September 2019. Throughout the period covered by this report, AFLP patients were treated in relatively uniform fashion by residents who were supervised by obstetrics faculty and fellows. Data of all patients were entered into a registry and retrieved for the study with approval of the two medical centers. All patients were followed up for 3 months. This work has been carried out in accordance with the Declaration of Helsinki (2000) of the World Medical Association. The study was approved by the Ethics Committee of Xiangya Hospital Central South University. All participants provided written informed consent.

Diagnosis of AFLP was made on the basis of both clinical and laboratory criteria and Swansea criteria [15, 16]. Clinical and laboratory criteria were as follows: (i) patients with symptoms of anorexia, fatigue, nausea, vomiting, jaundice and abnormal liver function in the third trimester of pregnancy; (ii) characteristic laboratory examination; (iii) ultrasound imaging showing fatty liver; (iv) viral hepatitis, pharmaceutic hepatitis, toxic hepatic and other hepatic diseases associated with pregnancy were excluded by clinical feature and laboratory examination; and (v) liver biopsy in accordance with pathologic changes. While the Swansea criteria were as follows. Patients with six or more of the following features in the absence of any other explanation for their symptoms were diagnosed with AFLP: Vomiting; Abdominal pain; Polydipsia/polyuria; Encephalopathy; Elevated bilirubin (>14µmol/L); Hypoglycaemia (<4mmol/L); Elevated urate (>340µmol/L); Leucocytosis (>11 × 10^9^/L); Ascites or bright liver on ultrasound scan (USS); Elevated transaminases (Alanine Transaminase/Aspartate Aminotransferase>42IU/L); Elevated ammonia (>47µmol/L); Renal impairment (Creatinine>150umol/L); Coagulopathy (prothrombin time>14s or activated partial thromboplastin time>34s); Microvesicular steatosis on liver biopsy.

We analysed clinical and laboratory variables from admission to one week after delivery to identify predictive factors of mortality in AFLP patients. Clinical variables that closely related to outcome of AFLP were maternal age, gestational age, referral situation, parity, delivery mode, twin pregnancy, interval time from AFLP attack to pregnancy termination, serious complications and artificial liver support (ALS) treatment or not. The indication for ALS treatment of our study was for patients with severe AFLP. Patients with any of the following features were identified as severe AFLP: total bilirubin (TBIL)>171µmol/L; prothrombin time activity (PTA) < 40%; hepatorenal syndrome; and hepatic encephalopathy. ALS treatment was promptly administered after pregnancy termination, subject to the consent of both the patients and their families if deemed appropriate. Laboratory variables that indicated AFLP were INR, white blood cell count (WBC), haemoglobin, haematocrit (HCT), platelet counts (PLT), TBIL, alanine aminotransferase (ALT), Aspartate Aminotransferase (AST), serum creatinine (Scr), blood glucose, blood urea nitrogen, blood ammonia, blood urate, lactate, serum sodium (Na) and serum calcium (Ca). Univariate and multivariate cox regression analyses were used to identify predictive factors of AFLP related mortality. Due to the small sample size of outcome events and the numerous prognostic factors associated with AFLP in this study, we initially conducted a univariate Cox regression analysis to eliminate irrelevant independent variables, followed by a multivariate Cox regression analysis to ensure the robustness of our findings.

MELD, MELD-Na, iMELD and PSLD scores were calculated as follows:

MELD score [4] = 9.57 × Log_e_ (creatinine [mg/dL]) + 3.78 × Log_e_ (bilirubin [mg/dL]) + 11.20 × Log_e_ (INR) + 6.43× (etiology: 0 if cholestatic or alcoholic, 1 otherwise).

MELD-Na score [8] = MELD – Na [mmol/l] − [0.025 × MELD × (140 − Na [mmol/l]) + 140.

iMELD score [10] = MELD + (0.3 × age [years]) − (0.7 × Na [mmol/l]) + 100.

PSLD score [11] = (1.17 × total bilirubin [mg/dl]) + (2.09 × INR).

The area under the receiver operating characteristic curve (AUC) was used to compare prognostic efficiency of different models.

All statistical analyses were conducted using the SPSS 18.0 (IBM, Armonk, NY, USA) statistical software program. Continuous variables were expressed as means and standard deviations, tested for normal distribution, assessed with *t*-tests and variables not normally distributed were assessed by the Wilcoxon’s rank-sum test. Non continuous variables were presented as median and range. Categorical variables were assessed with chi-square tests. Time-covariate test was used to assess proportional hazards assumes. Univariate analysis of risk factors for death was performed using the Cox proportional hazard model, and covariates with *P* ≤ 0.10 were considered significant (to avoid eliminating significant variables). Variables found to be significant on univariate analysis were included in a multivariate Cox regression analysis, performed using a likelihood ratio (LR) approach, *P* ≤ 0.10 (two-tailed) was considered statistically significant.

## Results

There were 99 consecutive patients who were highly suspected to have AFLP admitted to our centers. All patients were separately diagnosed using both the clinical and laboratory criteria and the Swansea criteria. Among them, 3 patients were excluded for incomplete data. For the remaining 96 patients, cases complicated with hepatitis B virus infection (n = 6) and HELLP syndrome (n = 2) were eliminated. And 10 cases were excluded because they did not satisfy six Swansea Criteria for diagnosis of AFLP. Thus, a total of 78 confirmed cases fulfilled both clinical and laboratory criteria and Swansea Criteria were included in our study. All cases were assessed by at least three professors.

After three months of follow-up, 11 of the 78 patients died (14.10%) in our study. Most deaths occurred due to encephalopathy, multi-organ failure, postpartum haemorrhage, disseminated intravascular coagulation or severe infection. 25 patients were transferred to our centers after delivery. The three-month mortality rate of transferred patients was 16% (4/25). Of the 53 patients admitted to our centers before delivery, 49 cases terminated gestation within 24 h after admission. Mean maternal age of the 78 cases was 27 years (range 19–41 years) and mean gestational age at delivery was 35.55 weeks (range 28–42 weeks). The perinatal mortality of the 78 cases was 17.95% (14/78).

Clinical and laboratory data obtained from admission to one week after delivery were used for analysis. First, we put laboratory data obtained before delivery into univariate analysis, differences in laboratory data between the survivors and non-survivors have been summarised in Table 1. Univariate analysis showed that mortality was significantly related to HCT and lactate.

**Table 1:** Univariate analysis of laboratory variables influencing maternal mortality on admission

Variables	Range	Alive (n = 67) Mean ± SD	Dead (n = 11) Mean ± SD	*P* value (< 0.1)
INR	1.07-10.0	2.13 ± 1.44	2.11 ± 0.85	0.976
Total bilirubin (µmol/l)	20.8-366.4	154.33 ± 90.57	210.10 ± 100.15	0.140
ALT(U/L)	83–886	236.72 ± 176.15	172.24 ± 121.59	0.356
AST(U/L)	28.4-800.6	270.31 ± 165.05	193.64 ± 111.67	0.242
BUN(mmol/L)	0.62–21.13	7.88 ± 3.90	8.07 ± 3.88	0.907
Creatinine (µmol/l)	35.6-281.3	175.25 ± 64.74	194.76 ± 34.10	0.441
Serum sodium (mmol/l)	121-149.6	133.54 ± 5.95	132.49 ± 4.24	0.654
Serum calcium (mmol/l)	1.63–2.67	2.13 ± 0.26	2.17 ± 0.26	0.699
WBC count (×10^9^/l)	5.7–35.9	15.11 ± 5.70	15.24 ± 5.36	0.957
HGB(g/l)	48–155	110.67 ± 23.83	106.71 ± 23.52	0.683
PLT(×10^9^/l)	41–317	141.60 ± 63.91	156.14 ± 63.54	0.578
Haematocrit (%)	16.8–48.0	33.81 ± 7.31	28.44 ± 6.95	0.077
Serum glucose (mmol/l)	2.15–11.2	5.08 ± 2.03	4.33 ± 1.55	0.356
Ammonia (µmol/l)	2.09–163.1	51.43 ± 29.38	84.77 ± 48.45	0.157
Lactate(mmol/l) ^a^	0.9–10.5	2.85 ± 2.43	6.11 ± 3.59	0.056
Urate (µmol/l)	221.2-1145.1	555.44 ± 198.06	610.40 ± 133.35	0.484
ALP(U/L)	52.2-1164.5	431.36 ± 285.39	362.12 ± 298.89	0.616
LDH(U/L)	214.6-2665.4	709.04 ± 410.03	924.39 ± 279.43	0.288

SD: standard deviation, INR: international normalised ratio, ALT: Alanine Transaminase, AST: Aspartate Aminotransferase, BUN:blood urea nitrogen, WBC:white blood cell count, PLT:platelet counts, ALP: Alkaline Phosphatase, LDH: lactate dehydrogenase

Notes: a There were 18 alive cases and 5 dead cases monitored the blood lactate level

To further detect prognostic factors for outcome and explore patients’ reaction to delivery and treatment, we put the data obtained at 72 h postpartum into univariate analysis. Differences in clinical and laboratory data between the survivors and non-survivors have been summarised in Table 2. All variables in cox regression analysis were confirmed to proportional hazards assume. Univariate analysis showed that mortality was significantly related to INR (*P* = 0.014), TBIL (*P* = 0.035), Scr (*P* = 0.018), lactate (*P* = 0.035), PLT (*P* = 0.024) and hepatic encephalopathy (*P* = 0.035). Then we put INR, TBIL, Scr, PLT and hepatic encephalopathy into multivariate Cox regression analysis. Factors such as postpartum haemorrhage, ALS, twin pregnancy, parity, gestational age, perinatal death, which were not detected in the univariate analysis but thought to be important in clinical, were also included in multivariate analysis. INR (HR = 3.446; 95% CI, 1.324–8.970, *P* = 0.011), TBIL (HR = 1.005; 95% CI, 1.000-1.010, *P* = 0.064), Scr (HR = 1.007; 95% CI, 1.001–1.013, *P* = 0.017), PLT (HR = 0.964; 95% CI, 0.931–0.997, *P* = 0.035) and ALS (HR = 0.123; 95% CI, 0.012–1.254, *P* = 0.077) were revealed as independent prognostic factors for poor AFLP outcomes. (Table 2) In our study, a total of 55 patients were identified as severe cases, out of which 16 patients were selected for ALS treatment while the remaining patients underwent conservative treatment. During both univariate and multivariate analysis, ALS was exclusively analyzed within these 55 patients. Among the 16 cases who received ALS treatment, 6 underwent plasma exchange, 3 received hemodialysis and continuous renal replacement therapy, another 3 underwent molecular adsorbent recirculating system (MARS) treatment, and finally, 4 cases received a combination of plasma exchange and MARS. Unfortunately, among the group that underwent ALS treatment, there were two patient fatalities.

**Table 2:** Univariate and multivariate analysis of factors influencing maternal mortality at 72 h postpartum

Variables	Alive(n = 67)	Dead(n = 11)	Univariate Analysis		Multivariate Analysis	
			HR (95% CI)	P(< 0.10)	HR (95% CI)	P(< 0.10)
Age (years)	26.95 ± 5.91	25.73 ± 3.85	0.97(0.87–1.07)	0.47		
Gestational age (weeks)	35.48 ± 2.66	35.90 ± 2.75	1.05(0.81–1.35)	0.72	0.85(0.63–1.15)	0.30
Multiparity	24	2	0.42(0.09–1.92)	0.26		
Twin pregnancy	22	3	0.79(0.21–2.99)	0.73	1.47(0.15–14.43)	0.74
Perinatal death	12	2	1.04(0.22–4.80)	0.96	0.26(0.02–3.05)	0.29
Referral after delivery	21	4	1.18(0.35–4.02)	0.79		
Vaginal delivery	18	0	0.03(0.00-8.21)	0.22		
Postpartum hemorrhage	24	5	1.53(0.47–5.01)	0.48	1.71(0.28–10.54)	0.57
Hepatic encephalopathy	15	6	3.59(1.10-11.77)	0.04	2.88(0.43–19.53)	0.28
artificial liver support^a^	14	2	0.47(0.06–3.64)	0.47	0.12(0.01–1.25)	0.08
USS^b^	40	9	0.92(0.20–4.26)	0.92		
INR	1.78 ± 0.67	2.33 ± 0.86	1.99(1.15–3.44)	0.01	3.45(1.32–8.97)	0.01
Total bilirubin (µmol/l)	185.44 ± 114.95	266.66 ± 166.16	1.01(1.00-1.01)	0.04	1.01(1.00-1.01)	0.06
ALT(U/L)	83.46 ± 127.01	41.22 ± 14.80	0.98(0.95–1.01)	0.12		
AST(U/L)	85.44 ± 108.62	79.11 ± 37.40	1.00(0.99–1.01)	0.88		
Creatinine (µmol/l)	171.79 ± 110.76	263.32 ± 106.77	1.00(1.00-1.01)	0.02	1.01(1.00-1.01)	0.02
Serum sodium (mmol/l)	135.11 ± 5.47	134.07 ± 10.03	0.97(0.89–1.06)	0.53		
Serum calcium(mmol/l)	1.92 ± 0.17	1.90 ± 0.24	0.70(0.03–19.08)	0.83		
WBC count (×10^9^/l)	20.22 ± 7.16	21.06 ± 6.21	1.01(0.93–1.10)	0.74		
haemoglobin (g/l)	86.83 ± 18.81	85.45 ± 26.30	1.00(0.97–1.03)	0.81		
platelet (×10^9^/l)	99.49 ± 61.39	58.64 ± 23.20	0.98(0.96 − 1.00)	0.02	0.96(0.93-1.00)	0.04
Haematocrit (%)	26.16 ± 5.24	25.45 ± 7.33	0.98(0.87–1.09)	0.69		
Serum glucose (mmol/l)	4.50 ± 2.25	5.32 ± 1.75	1.06(0.82–1.36)	0.68		
BUN(mmol/L)	11.77 ± 6.84	13.94 ± 7.99	1.03(0.96–1.12)	0.40		
Ammonia (µmol/l)	46.52 ± 25.18	52.48 ± 25.15	1.01(0.99–1.03)	0.40		
Urate (µmol/l)	429.61 ± 196.53	529.22 ± 329.80	1.00(1.00–1.00)	0.17		
ALP(U/L)	306.43 ± 193.63	302.37 ± 271.53	1.00(1.00–1.00)	0.89		
LDH(U/L)	651.38 ± 432.54	777.49 ± 697.83	1.00(1.00–1.00)	0.56		
Lactate(mmol/l)^c^	3.01 ± 2.34	5.79 ± 3.11	1.40(1.03–1.91)	0.04		

Continuous variables are shown as mean ± standard deviation (SD). HR: hazard ratio, CI: confidence interval; USS: Ascites or bright liver on ultrasound scan, INR: international normalised ratio, BUN: blood urea nitrogen, WBC:white blood cell count, PLT:platelet counts, ALT: Alanine Transaminase, AST: Aspartate Aminotransferase, ALP: Alkaline Phosphatase, LDH: lactate dehydrogenase

Notes: a A total of 55 patients were identified as severe cases, artificial liver support was exclusively analyzed within these 55 patients

b There were 48 alive cases and 11 dead cases received ultrasound scan

c There were 18 alive cases and 5 dead cases monitored the blood lactate level

The cox regression model was h (t) = h_0_(t) exp (1.237×INR + 0.005×TBIL + 0.007×Scr-0.037×PLT-2.096×ALS) and fitted well with likelihood χ2 = 21.0, *P* = 0.001. The prognosis index (PI) for AFLP (PI-AFLP) was calculated as follows: PI = 1.237×INR + 0.005×TBIL + 0.007×Scr-0.037×PLT-2.096×(ALS: 0 without ALS treatment, 1 with ALS treatment). Patients were in high risk of death with a high PI score. Predictive accuracy of PI-AFLP, as measured using the receiver operating characteristic (ROC) curve, was 0.874. A cut-off PI-AFLP score of 2.98 yielded 72.7% sensitivity and 89.1% specificity. Inconsistency rate of PI-AFLP was 11.54% (9/78).

**Fig. 1:**
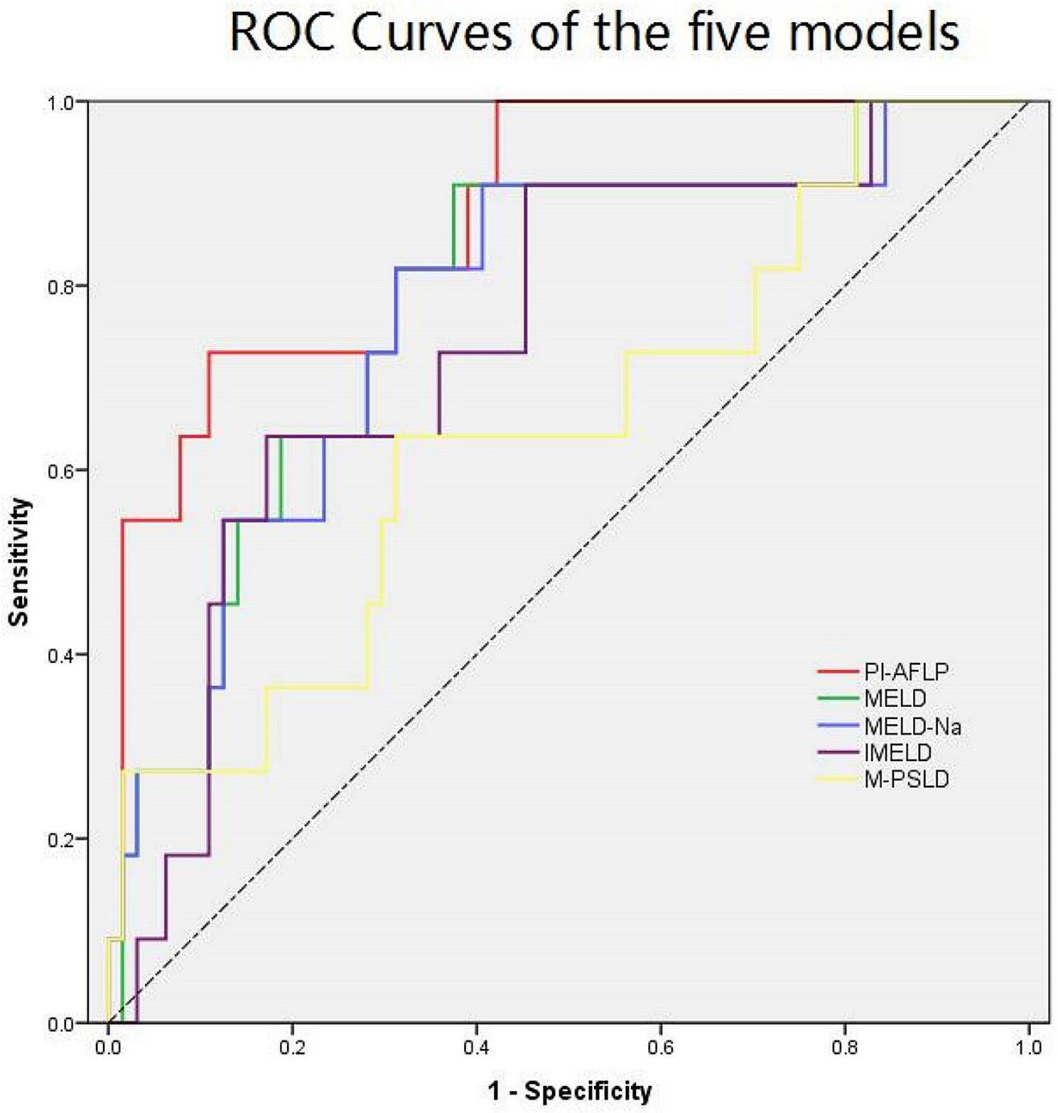
Comparison of accuracy of five models to predict three-month mortality of AFLP. Note: The area under the receiver operating characteristic curve (AUC) was showed on the receiver operating characteristic (ROC) curve. The PI-AFLP (red) had the largest AUC, followed by the MELD (green), MELD-Na (blue), iMELD (purple) and M-PSLD (yellow)

Then, to further study the efficacy and accuracy of the new prognostic model, we compared PI-AFLP model with MELD, MELD-Na, iMELD and PSLD models in predicting outcomes of AFLP, using ROC curve. The AUCs of the PI-AFLP, MELD, MELD-Na, iMELD and PSLD models were 0.874,0.781,0.774,0.744 and 0.643, respectively (Table 3; Fig. 1), and the inconsistency rate were 11.54%, 26.92%, 26.92%, 33.33%, 30.77%, respectively (Table 3).

**Table 3:** Comparison of accuracy and inconsistency of 5 models in predicting prognosis of patients with AFLP

Model	AUC	Cut-off	Sensitivity	Specificity	Inconsistency rate	95% CI for AUC
PI-AFLP	87.4%	2.98	72.7%	89.1%	11.54%	0.769–0.978
MELD	78.1%	30.37	72.7%	71.9%	26.92%	0.636–0.926
MELD-Na	77.4%	32.09	72.7%	71.9%	26.92%	0.624–0.925
iMELD	74.4%	43.93	72.7%	64.1%	33.33%	0.591–0.898
M-PSLD	64.3%	20.93	63.6%	68.7%	30.77%	0.459–0.828

PI-AFLP: prognostic index for acute fatty liver of pregnancy; MELD: Model for End Stage

Liver Disease; MELD-Na: Model for end-stage liver disease with the incorporation of serum sodium; iMELD: Integrated model for end-stage liver disease; M-PSLD: Model to predict mortality of pregnancy-specific liver diseases; AUC: Area under the receiver operating characteristic curve

Finally, we depicted recovery of INR, TBIL, Scr and PLT from delivery to one week after delivery in survivors and non-survivors. In survivors, INR began to decline 3–4 days after delivery, TBIL started to decrease 4–5 days after delivery, Scr started to decrease 2–3 days after delivery, and PLT began to recover 5–6 days after delivery. While in non-survivors, INR, TBIL, Scr and PLT delayed restoration or non-restoration.

## Discussion

We adopted both clinical and laboratory criteria and Swansea criteria to confirm diagnosis of cases included in this study. Meanwhile, all cases were reviewed by at least three professors to determine the diagnosis. The clinical and laboratory criteria, proposed by Ze-yi CAO [15], was widely regarded as an objective and sensitive method for diagnosis of AFLP in China. The Swansea criteria, proposed by Ch’ng CL [16], had been validated as a good screening tool with positive and negative predictive value of 85% and 100% for hepatic microvesicular steatosis tested by liver biopsy [17]. Since almost 90% of the AFLP patients were associated with coagulation dysfunction and liver biopsy was contraindicated, Swansea criteria combined with clinical assessment was the most common and practical method for diagnosis of AFLP. In addition to this, there is one more thing to note that confirmation by known mutations like fetal homozygous mutation (1528G > C) in the gene that encodes for mitochondrial long-chain hydroxy acyl-CoA dehydrogenase (LCHAD) can also confirm the diagnosis in part of the patients, although often no known mutation is found [18].

Previous studies on the prognosis of AFLP have mostly analysed prenatal laboratory data [19, 20], and there are rare studies about correlation between postnatal laboratory indicators and AFLP prognosis. This paper also planned to study antenatal laboratory index on the prognosis of AFLP at the beginning, and results showed that HCT and lactate levels were associated with poor prognosis. However, the results differed greatly from previous studies, and we reflected on that this may be due to the prenatal laboratory data tested by different laboratories and laboratory standards of 25 patients who were transferred to our hospitals after delivered from local hospitals. Because the reference and test method were not consistent, bias may be produced. Therefore, we further studied the prognosis of AFLP using laboratory data tested 72 h after delivery in the same laboratory method to check the results of the study. Interestingly, we found that INR, TBIL, Scr, PLT, lactate and hepatic encephalopathy were highly related to mortality of AFLP. INR, TBIL and Scr had been revealed to be prognostic factors for both end stage liver disease and PSLD [4–11], and also appear to be useful in AFLP. Thrombocytopenia and Scr were found to be independent risk factors for postpartum complications occurred in pre-eclampsia and HELLP syndrome such as infection, disseminated intravascular coagulation, acute renal failure and needing for blood transfusion [21]. These complications can be also occurred in AFLP patients. Studies have shown that lactate was an independent risk factor for poor prognosis in patients with liver failure and cirrhosis [22, 23]. Lactate combined with hepatic encephalopathy had been proved to have a 90% sensitivity to predict which patients with acute liver failure in pregnancy are likely to die or require transplantation [24]. Regrettably, a further study about lactate and prognosis was limited as lactate was not routinely monitored in AFLP patients in our centers. Only 23 of 78 patients were monitored blood lactate level in our study.

Then, multivariate Cox regression analysis further revealed INR, TBIL, Scr, PLT and ALS as significant variables. ALS is an external mechanical, chemical or biological device to temporarily or partially replace liver function, so as to assist in the treatment of liver insufficiency, liver failure or related diseases. It was proved to be an effective method to improve outcome of AFLP [25, 26]. The commonly used artificial liver treatment methods include plasma exchange, hemofiltration, hemoperfusion, plasma perfusion, MARS, etc. Timely application of this treatment in the early phase of severe AFLP patients can effectively halt and reverse disease progression. Fitting time of ALS can protect liver cells from reducing mitochondrial damage due to oxidative stress and gain chance to improve patients’ prognosis [25]. PI-AFLP, calculated with INR, TBIL, Scr, PLT and ALS, was found to be able to accurately identify AFLP patients’ poor outcomes with an AUC of 87.4%, a sensitivity of 72.7% and a specificity of 89.1%. Our results highlighted the role of elevated INR, TBIL, Scr, and low PLT as significant factors for mortality of AFLP. However, due to the limited outcome event (death) in this study, the prediction of mortality by this model needs to be verified with a larger sample size and outcome event size (death) in a future study. In order to further determine the predictive effect of these factors on AFLP mortality, we tracked the changes of corresponding factors after delivery, and found that INR, TBIL, Scr and PLT counts delayed restoration or non-restoration in non-survivors. This further confirmed our hypothesis.

Furthermore, we compared prognostic efficiency of PI-AFLP with MELD, MELD-Na, iMELD and PSLD models in predicting AFLP outcomes. PI-AFLP has the highest efficiency for predicting mortality of AFLP. That’s because this PI was developed specifically to predict mortality among AFLP patients. In contrast, MELD, MELD-Na and iMELD were developed to predict mortality in end-stage liver disease and didn’t consider the influence of pregnancy. PSLD model was developed for multiple liver diseases during pregnancy and may not be pertinent to AFLP.

We also assessed inconsistencies between predicted and actual outcome for PI-AFLP model. The inconsistency rate was 11.54% (9/78). Among them, 7 cases were wrongly judged to be dead, and 2 cases were wrongly judged to be alive. It showed that the new prediction model was biased towards heavy judgment, which was helpful for early identification of critical patients and taking timely intervention to improve patients’ prognosis.

However, our study has some limitations. First, some patients had different clinical conditions. 25 patients were transferred to our hospitals after delivery, treatment among different rank of hospitals may result in different intervene. Second, because of the retrospective design, we could have missed some data. This limitation may be balanced by relatively large cases. Third, small sample size and limited event (death) may lead to instability of the prediction model. Future studies should consider increasing large numbers of cases or conducting multi-center studies to address this limitation. What’s more, external validation of the model is required in patients around the world before generalisability can be assumed.

## Conclusions

In summary, our study demonstrates that INR, TBIL, Scr and PLT at 72 h postpartum are significant variables in predicting mortality of AFLP. ALS is an effective measure to improve severe patients’ prognosis. PI-AFLP calculated by TBIL, INR, Scr, PLT and ALS was a sensitive and specific model to predict mortality of AFLP. Further prospective studies across all geographical regions are needed to develop a more accurate and unique model for prognosis of AFLP.

## Data Availability

The datasets used and analyzed during the current study are available from the corresponding author on reasonable request.

## Abbreviations

AFLP: Acute fatty liver of pregnancy
ALS: Artificial liver support
ALT: Alanine Transaminase
ALP: Alkaline Phosphatase
APACHE II: Acute Physiology and Chronic Health Evaluation II
AST: Aspartate Aminotransferase
AUC: Area under the receiver operating characteristic curve
BUN: Blood urea nitrogen
CI: Confidence interval
HCT: Haematocrit
HELLP syndrome: Haemolysis, elevated liver enzymes and low platelet count syndrome
HR: Hazard ratio
iMELD: Integrated model for end-stage liver disease
INR: International normalised ratio
LCHAD: Long-chain hydroxy acyl-CoA dehydrogenase
LDH: Lactate dehydrogenase
LR: Likelihood ratio
MARS: Molecular adsorbent recirculating system
MELD: Model for End Stage Liver Disease
MELD-Na: Model for end-stage liver disease with the incorporation of serum sodium
PSLD: Pregnancy-specific liver diseases
PTA: Prothrombin time activity
PI-AFLP: Prognostic index for acute fatty liver of pregnancy
PLT: Platelet counts
ROC: Receiver operating characteristic
Scr: Serum creatine
SOFA: Sequential Organ Failure Assessment
TBIL: Total bilirubin
USS: Ascites or bright liver on ultrasound scan
WBC: White blood cell count
